# In Vitro Chemopreventive Potential of Phlorotannins-Rich Extract from Brown Algae by Inhibition of Benzo[a]pyrene-Induced P2X7 Activation and Toxic Effects

**DOI:** 10.3390/md19010034

**Published:** 2021-01-14

**Authors:** Mélody Dutot, Elodie Olivier, Sophie Fouyet, Romain Magny, Karim Hammad, Emmanuel Roulland, Patrice Rat, Roxane Fagon

**Affiliations:** 1 Recherche & Développement, Yslab, 29000 Quimper, France; roxane.fagon@yslab.fr; 2 Faculté de Pharmacie de Paris, UMR CNRS 8038, Université de Paris, 75006 Paris, France; elodie.olivier@parisdescartes.fr (E.O.); sophie.fouyet@etu.parisdescartes.fr (S.F.); romain.magny@inserm.fr (R.M.); karim.hammad@parisdescartes.fr (K.H.); emmanuel.roulland@parisdescartes.fr (E.R.); patrice.rat@parisdescartes.fr (P.R.)

**Keywords:** A549 cells, cancer, cytoskeleton, phlorotannins, P2X7 receptor, pollution

## Abstract

Phlorotannins are polyphenols occurring exclusively in some species of brown algae, known for numerous biological activities, e.g., antioxidant, antiproliferative, antidiabetic, and antiallergic properties. Their effects on the response of human lung cells to benzo[a]pyrene (B[a]P) has not been characterized. Our objective was to in vitro evaluate the effects of a phlorotannin-rich extract obtained from the brown algae *Ascophyllum nodosum* and *Fucus vesiculosus* on B[a]P cytotoxic effects. The A549 cell line was incubated with B[a]P for 48 and 72 h in the presence or absence of the brown algae extract. Cytochrome P450 activity, activation of P2X7 receptor, F-actin disorganization, and loss of E-cadherin expression were assessed using microplate cytometry and fluorescence microscopy. Relative to control, incubation with the brown algae extract was associated with lower B[a]P-induced CYP1 activity, lower P2X7 receptor activation, and lower reactive oxygen species production. The brown algae extract inhibited the alterations of F-actin arrangement and the downregulation of E-cadherin expression. We identified a phlorotannins-rich extract that could be deeper investigated as a cancer chemopreventive agent to block B[a]P-mediated carcinogenesis.

## 1. Introduction

Humans can be exposed to polycyclic aromatic hydrocarbons (PAHs) occurring in the environment, at home, and in the workplace. Because PAHs exist naturally in the environment and are man-made, exposure occurs in a number of ways. Fumes from vehicle exhaust, coal, coal tar, asphalt, wildfires, agricultural burning and hazardous waste sites are all sources of exposure. The largest emission of PAHs globally comes from incomplete combustion of organic material, and the largest single source is from the combustion of biofuels. PAHs are a major health concern because they remain in the environment for long periods of time, and are therefore persistent pollutants, and represent important air pollutants. Big cities around the world face severe atmospheric pollution problems, which directly affects the population’s health. In this context, sixteen PAHs are regulated by the U.S. Environmental Protection Agency (USEPA) based on their potential human and ecological health effects. Benzo[a]pyrene (B[a]P) is considered as representative of the whole PAH group and is consequently the best-investigated single PAH compound [1]. To date, B[a]P is the only PAH classified in group 1 by the International Agency for Research on Cancer (IARC) and is a known carcinogen to humans [2]. B[a]P is toxic by dermal and oral exposure, and mainly by inhalation. As one of the most common ways B[a]P can enter the human body is through breathing contaminated air, B[a]P is responsible for many respiratory disorders, such as lung cancer and asthma [3,4], and can aggravate allergic rhinitis [5].

B[a]P is metabolically activated by phase I P450 isozymes, mainly CYP1A1 and CYP1B1, into epoxides that can induce DNA damages. In epithelial cells, B[a]P and its metabolites promotes tumor invasion and metastasis through the epithelial-mesenchymal transition (EMT) [6]. During EMT, epithelial cells lose their epithelial functions and characteristics causing the enhancement of mobility and invasiveness. 

P2X7 receptor is abundantly expressed in cancer cells of leukemia, neuroblastoma, melanoma, as well as in prostate, breast and thyroid cancer [7,8,9,10,11], and has been proposed to be a biomarker for early stage cancer [9,12,13]. Furthermore, activation of P2X7 was reported to have anti-apoptotic effects, stimulate tumor cell growth [14,15], and even to promote cell invasiveness in some cancer cells [16,17], which is contradictory to the initial assumption that P2X7 was a death receptor. In cancer cells, P2X7 seems to be uncoupled from intracellular cell death-promoting pathways [10]. An autocrine ATP-P2X7 signaling is suggested to be involved in migration of human lung cancer cells through regulation of actin cytoskeleton rearrangement [18]. We previously demonstrated that B[a]P induces P2X7 receptor activation on human placental JEG-3 cells [19] but the role of P2X7 in B[a]P toxicity for lung cells has not been published. 

Marine-sourced algae are a varied source of pharmacologically vital natural products. Among them, phlorotannins are unique polyphenolic compounds, which are not found in terrestrial plants, but only in some brown algal species; *Ascophyllum nodosum* and *Fucus vesiculosus* are one of the richest sources of phlorotannins [20]. Phlorotannins result from the tridimensional polymerization of phloroglucinol and possess potent antioxidant and anti-inflammatory activities [21,22]. It has been proposed that the radical scavenging activity of phlorotannins can be used indirectly to reduce cancer formation in human body [23,24]. To the best of our knowledge, the phlorotannins’ effects on P2X7 activation induced by B[a]P have not previously been characterized. The aim of this work was to assess the modulation of B[a]P toxic effects on pulmonary human A549 cells using a brown algae extract rich in phlorotannins. B[a]P metabolism, P2X7 receptor activation, filament actin arrangement, and E-cadherin expression were studied with and without preincubation with the algal extract prior to B[a]P stimulation.

## 2. Results

### 2.1. Cytochrome P450 Activity

**Figure 1:**
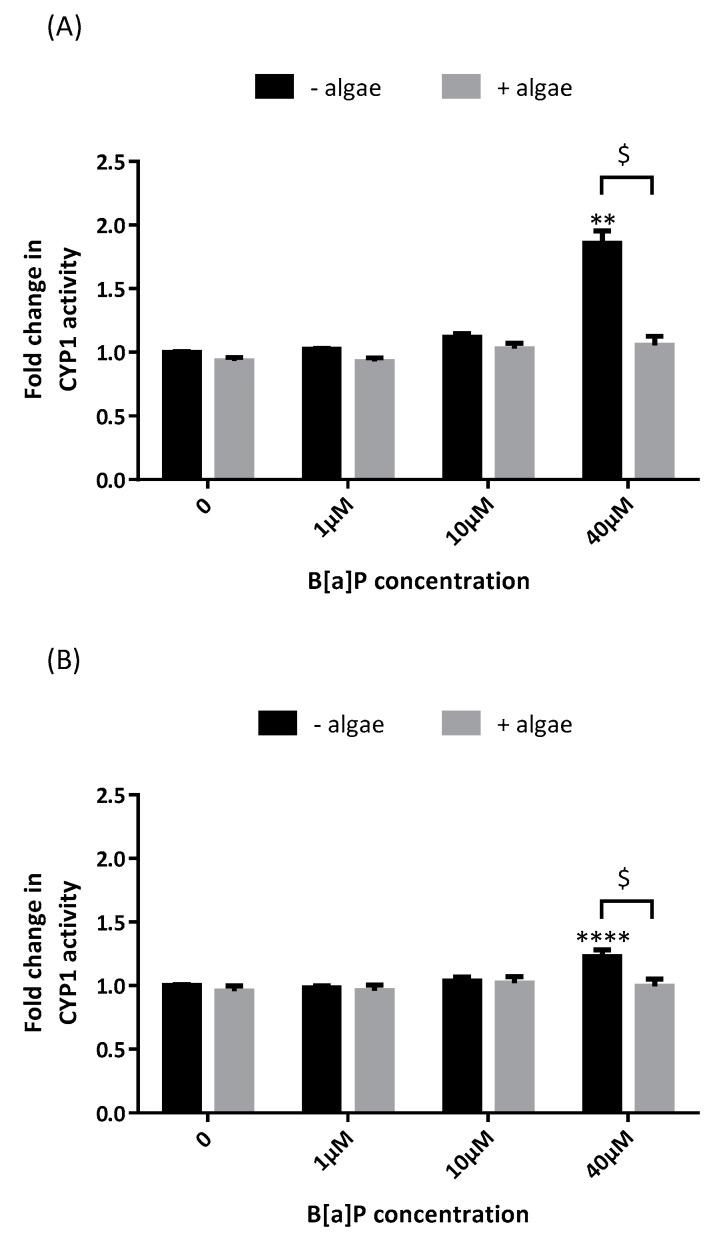
Effects of the brown algae extract on the P450 1-dependent monooxygenases of A549 cells incubated with different concentrations of B[a]P (ethoxyresorufin-O-deethylase (EROD) assay). Cells were incubated with either phosphate buffered saline PBS (control, black bars) or the brown algae extract at 0.1% (grey bars) for 20 min. The solutions were then removed, and the cells were incubated with B[a]P for 48 h (**A**) or 72 h (**B**). Data correspond to the mean ± SEM of four independent experiments. The significance thresholds were ** p < 0.01, **** p < 0.0001 and $ *p* < 0.05.

The effects of the brown algae extract on B[a]P activation of the cytochrome P450 enzyme system were evaluated by assessment of ethoxyresorufin-O-deethylase (EROD) activity in A549 cells supplemented with the brown algae extract at 0.1% (Figure 1). As expected, B[a]P induced CYP1 activity at both 48 h (Figure 1A) and 72 h (Figure 1B). The fold increase in CYP1 activity compared to control was higher after 48 h than after 72 h (×1.9 and ×1.2, respectively). B[a]P did not induce CYP1 activity at neither 4 h nor 24 h (data not shown). The brown algae extract totally prevented CYP1 induction by B[a]P at both 48 h and 72 h.

### 2.2. P2X7 Receptor Activation

**Figure 2:**
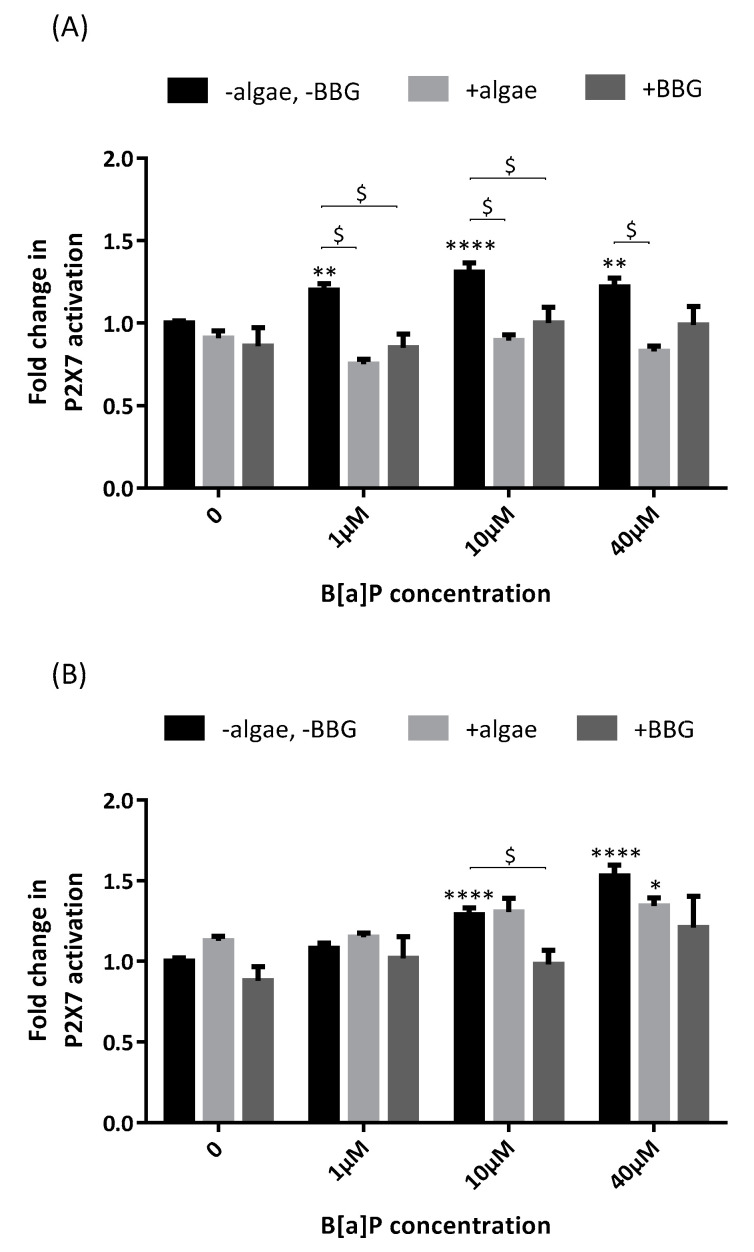
Effects of the brown algae extract on P2X7 receptor activation of A549 cells incubated with different concentrations of B[a]P (YO-PRO-1™ assay). Cells were incubated with either PBS (control, black bars) or the brown algae extract at 0.1% (light grey bars) or Brilliant Blue G (BBG) at 25 µM (dark grey bars) for 20 min. The solutions were then removed, and the cells were incubated with B[a]P for 48 h (**A**) or 72 h (**B**). Data correspond to the mean ± SEM of four independent experiments. The significance thresholds were * p < 0.05, ** *p* < 0.01, **** p < 0.0001, and $ *p* < 0.0001.

P2X7 receptor activation was significantly higher after B[a]P incubation compared to control at 48 h (×1.20 at 1 µM, ×1.27 at 10µM and ×1.22 at 40 µM, Figure 2A) and 72 h (×1.29 at 10 µM and ×1.53 at 40 µM, Figure 2B). The brown algae extract totally inhibited P2X7 activation when B[a]P was incubated for 48 h (×1.20 at 1 µM without the algae extract versus ×0.75 with the algae extract, ×1.27 versus ×0.89 at 10 µM and ×1.22 versus ×0.83 at 40 µM) but only partially when B[a]P was incubated for 72 h (×1.53 at 40 µM without the algae extract versus ×1.34 with the algae extract, not statistically significant). The brown algae extract was as efficient as the P2X7 antagonist Brilliant Blue G (BBG) in inhibiting P2X7 receptor activation by B[a]P after 48 h.

### 2.3. Actin Arrangement

**Figure 3:**
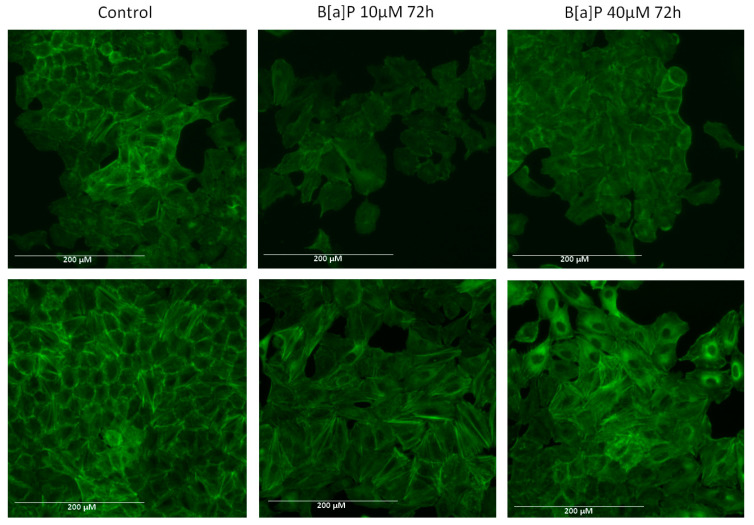
Fluorescence microscopic changes in F-actin cytoskeleton organization in A549 cell line. After B[a]P incubation, the cells were stained using ActinGreen 488 ReadyProbes^®^. The data shown are representative of three independent experiments. The images were captured under the same acquisition parameters by EVOS FL fluorescence microscope (Thermo Fisher Scientific). Upper panels: cells incubated in the absence of the brown algae extract, lower panels: cells incubated in the presence of the brown algae extract.

The actin cytoskeleton plays a crucial role in cellular movement and is strictly regulated during migration. Since cancer cell migration and metastasis requires reorganization of the cytoskeleton leading to EMT [25], we next addressed whether the brown algae extract could prevent B[a]P-induced reorganization of the components of the cytoskeleton. We picked the 72 h incubation time because changes were more obvious than after 48 h. Control cells have organized F-actin and long filaments are visible (Figure 3). Incubation with B[a]P induced a disruption of F-actin cytoskeleton arrangement and altered cell morphology (Figure 3, upper panels). Indeed, cells incubated with B[a]P compared to control cells showed more spreading and loss of elongated and polarized morphology. A preincubation for 20 min with the brown algae extract before a 72 h incubation time with B[a]P obviously protected actin structure against B[a]P-induced cytoskeleton disorganization (Figure 3, lower panels).

### 2.4. E-Cadherin Expression

**Figure 4:**
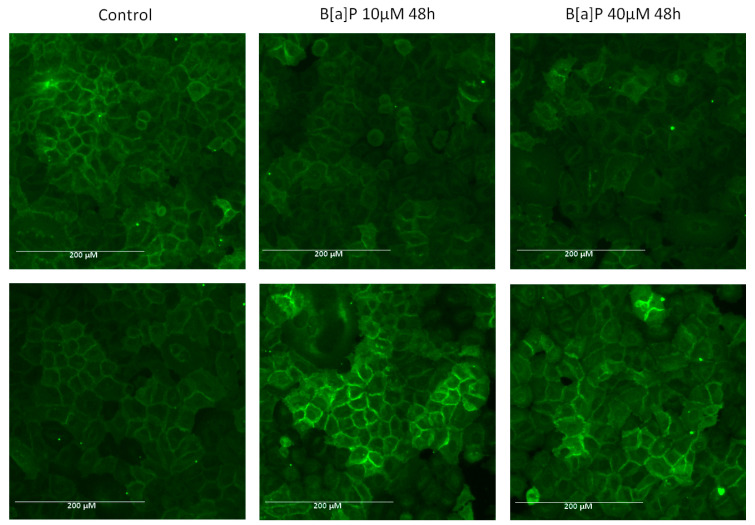
Immunofluorescence analysis of E-cadherin expression in A549 cells. Immunostaining was detected using antibody specific for E-cadherin and Alexa Fluor 488-labeled secondary antibody. The data shown are representative of three independent experiments. The images were captured under the same acquisition parameters by EVOS FL fluorescence microscope (Thermo Fisher Scientific). Upper panels: cells incubated in the absence of the brown algae extract, lower panels: cells incubated in the presence of the brown algae extract.

During EMT, the epithelial markers like E-Cadherin are suppressed. To determine if the brown algae extract can prevent B[a]P-induced epithelial markers alteration, we performed immunofluorescence staining of A549 cells with E-cadherin antibody. As shown in Figure 4 (upper panels), after A549 cells were incubated with B[a]P at 10 or 40 µM, protein expression of E-cadherin was abnormally low. However, the expression of E-cadherin was not altered when the cells were preincubated with the brown algae extract (Figure 4, lower panels).

### 2.5. Reactive Oxygen Species (ROS) Production

**Figure 5:**
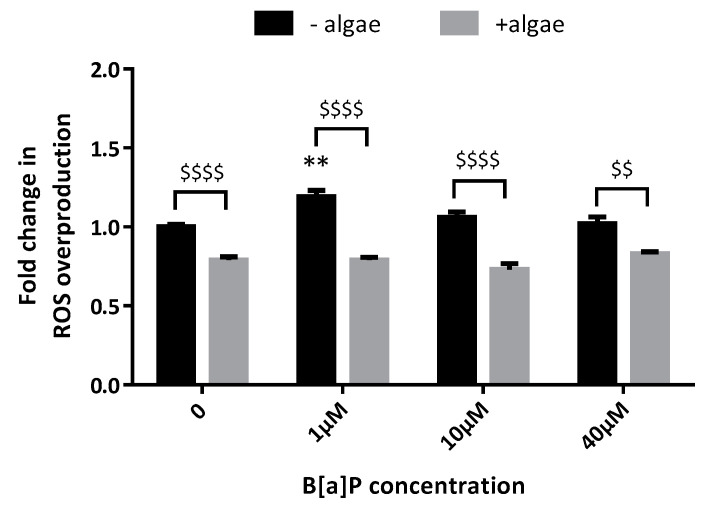
Effects of the brown algae extract on. reactive oxygen species (ROS) production by A549 cells incubated with different concentrations of B[a]P (H2DCF-DA assay). Cells were incubated with either PBS (control, black bars) or the brown algae extract at 0.1% (grey bars) for 20 min. The solutions were then removed, and the cells were incubated with B[a]P for 48 h. Data correspond to the mean ± SEM of four independent experiments. The significance thresholds were ** p < 0.01 and $$$$ *p* < 0.0001, $$ *p* < 0.01.

To better understand how the brown algae extract acts on A549 cells, subsequently we assessed ROS production after incubation with B[a]P. B[a]P 1 µM significantly elevated ROS production compared to control after 48 h (×1.19, Figure 5); that overproduction was totally inhibited by the brown algae extract. Whatever the B[a]P concentration used, the basal level of ROS production was significantly lower when the cells were incubated with the algae extract. B[a]P did not induce ROS overproduction after 72 h (data not shown). CYP1A1 and 1B1, CYP2C isoforms of ROS make them very challenging to detect. For example, the lifetime of singlet oxygen, superoxide, and hydroxyl radical in aqueous solutions is within the μs regime [26]. Libalova at al. has previously shown that BaP exposure has a very limited effect on ROS generation in A549 cells [27].

## 3. Discussion

The objective of the present study was to explore the effects of an algae extract from *Ascophyllum nodosum* and *Fucus vesiculosus* selected by the Yslab company on human pulmonary epithelial cells. A previous paper described the exact same extract and identified, apart from phlorotannins, the presence of fatty acids [28]. We confirmed by means of ^1^H-NMR that our batches are similar to theirs (Figures S1 and S2). 20 min of exposure to the algae extract was enough to inhibit the B[a]P-induced elevation in CYP1A activity, P2X7 receptor activation, actin rearrangement, E-cadherin depletion, and the production of reactive oxygen species by human pulmonary epithelial cells for 48 h and 72 h.

PAHs are ubiquitous pollutants and some of them are carcinogenic, B[a]P being the most carcinogenic PAH. Lungs are the main targets of PAHs in terms of tumor induction, we thus used cultured pulmonary cells to study the potential modulation of B[a]P toxic effects by a brown algae extract rich in polyphenols. A549 cells are metabolically active since they express both CYP1A1 and CYP1B1 and undergo formation of significant amounts of DNA adducts, they appear as a good model for lung toxicity of B[a]P [29,30].

Phlorotannins are abundant polyphenols found in brown algae. Over the last decade, phlorotannins purified from different species of brown algae have been more and more studied because of their broad spectrum of desirable biological activities, including antioxidant, antiviral, antiproliferative, radio protective, antidiabetic, skin protection, and antiallergic effects [31]. In an in vitro study of four pancreatic cancer cell lines, phlorotannins purified from the marine brown alga Fucus vesiculosus were found to (i) inhibit the growth of tumor cell lines and triggered apoptosis, (ii) induce cell fragmentation and cell cycle inhibition, and (iii) deregulate the expression of many genes involved in cell cycle control, DNA repair and cancer [32]. In pancreatic cells, the mechanisms underlying the phlorotannins’ anti-tumoral actions have yet to be elucidated. However, it has been reported that phlorotannins would influence autophagy that provides a rescue mechanism by stabilizing the cell metabolism [32,33].

The present study is the first to have demonstrated that the algae rich in phlorotannins (extracted from *Ascophyllum nodosum* and *Fucus vesiculosus*) also has protective effects on human pulmonary epithelial cells. In particular, the inhibition of the effects on pulmonary epithelial cells of B[a]P suggests that the algae rich in phlorotannins might dampen the epithelium’s toxic response to urban pollutants. In addition to the inhibition of CYP1 metabolic enzymes and P2X7 receptor activation, the inhibition of both actin rearrangement and E-cadherin depletion may have beneficial effect by reducing the risk of developing cancer. Further studies are needed to determine whether or not exposure to phlorotannins lowers lung carcinogenesis markers such as for example the expression of translationally controlled tumor protein (TCTP), tissue inhibitors of metalloproteinases-2 (TIMP-2), triosephosphate isomerase (TPI), or p53 [34,35]. In the meantime, it would be interesting to test phlorotannins-rich extracts from other brown algae that have been reported with health beneficial biological activities like *Undaria pinnatifida* and *Laminaria japonica*.

Among the various types of cancer chemopreventive agents are blocking agents, which inhibit the initiation stage of chemically induced carcinogenesis [36]. A blocking agent may act by various mechanisms [37]. As an example, it may inhibit the catalytic activity or suppress the expression of procarcinogen-bioactivating enzymes, such as the various cytochromes P450, including CYP1A1, CYP1A2, CYP1B1, and CYP2A6 [38]. Benzo[a]pyrene is a procarcinogen that undergoes CYP1-catalyzed bioactivation [39]. Therefore, we chose to determine the effect of the brown algae extract on benzo[a]pyrene metabolism using the EROD assay. The brown algae extract seems to be capable of inhibiting CYP1A1 and CYP1B1 enzymes induced by B[a]P at both 48 h and 72 h. It also inhibited P2X7 receptor activation but interestingly, the inhibiting effects were more potent after a 48 h incubation time with B[a]P. A positive feedback loop has been described in the literature, providing ATP-induced ATP release that can amplify the initial signal to provide a level of extracellular ATP high enough to re-activate P2X7 receptors [40]. The 20 min incubation time with the brown algae extract would not be long enough to counteract this amplification loop and repeated exposure to the extract could be more efficient to inhibit P2X7 receptor activation over the time. CYP1s were not activated after incubation with B[a]P at 1 µM and 10 µM, and yet we observed P2X7 receptor activation, cytoskeleton alteration, and decreased E-cadherin expression at those concentrations, which suggests that either B[a]P is metabolized through other mechanisms than CYP1 enzymes or B[a]P may be toxic by itself. Sulc et al. showed that CYP1A1 has the highest B[a]P-metabolizing potency, followed by CYP1B1, 2C19, and 3A4 to a lesser extent [41]. The A549 cells express CYP1A1 and 1B1, CYP2C isoforms and CYP3A4 [29,42,43]; B[a]P could therefore exert its P2X7 receptor inducing activity after metabolization by CYP2C and/or CYP3A4 enzymes. This hypothesis is rather unlikely since the metabolites generated by either CYP1, CYP2C, or CYP3A4 enzymes are the same, so the fold increase in P2X7 receptor activation after B[a]P at 40 µM (×1.22 at 48 h) should be higher than after B[a]P at 10 µM (×1.27) since the major metabolizing enzymes, namely CYP1 enzymes, are activated. B[a]P requires an enzymatic activation to exert its deleterious effects on pulmonary cells and apart from CYP1 metabolism, one pathway involves the formation of radical cations catalyzed by P450 peroxidases [44,45]. It seems that in our model, B[a]P could be toxic after both CYP1 and peroxidases metabolism through P2X7 receptor activation leading to cytoskeleton rearrangement and downregulation of the epithelial marker E-cadherin, two events occurring during EMT. The brown alga extract not only inhibited CYP1 activity, but also decreased P2X7 receptor activation and protected A549 cells against B[a]P-induced toxicity on F-actin and E-cadherin expression. After highlighting the antioxidant effects of the extract by measuring ROS production induced by B[a]P, we suggest that the algae extract could inhibit CYP1 and peroxidases and, thus, B[a]P metabolization. It has already been reported that chemopreventive properties of phlorotannins were due to their antioxidant capacity to inhibit the activity of glutathione peroxidase and CYP1A [46,47]. Besides, Barhoumi et al. showed that fatty acids affect enzyme activity related to phase I and phase II metabolism in B[a]P-incubated A549 cells [48]. The phlorotannin and fatty acids content of the extract could accordingly explain its modulating effects on CYP1 and P450 peroxidases in our model. Deeper B[a]P metabolism studies in the presence of the algae extract would help to better identify the enzymes it targets. In parallel, mRNA and/or protein investigations could be performed to study the specific effects of phlorotannins on the expression of CYP450 cytochromes and P2X7 receptor.

In conclusion, the results of our study suggest that the algae extract could be used to prevent or treat B[a]P-induced airway toxicity through P2X7 receptor inhibition and could be considered as a potential cancer chemopreventive agent to block B[a]P-mediated carcinogenesis.

## 4. Materials and Methods

Reagents: chemicals, including B[a]P, dimethyl sulfoxide (DMSO), EROD, Tween 20 and Triton X-100 were obtained from Merck (Darmstadt, Germany). Fluorescent dye YO-PRO-1™ was purchased from Thermo Fisher Scientific (Waltham, MA, USA). Cell culture reagents were purchased from Gibco (Paisley, UK).

Brown algae extract rich in phlorotannins: Yslab purchased the polyphenol-rich extract from innoVactiv (Rimouski, QC, Canada). According to the supplier, wild *A. nodosum* and *F. vesiculosus* brown algae were harvested from the North Atlantic Ocean where F. vesiculosus naturally occurs immediately above the *A. nodosum*. The extract was obtained from *A. nodosum* and *F. vesiculosus* in a ratio of 95/5.They were added to a crushing tank and crushing was followed by extraction using hot water as the only solvent. Solids were then separated from the algal juice by sifting. The algal juice was further processed to partially remove minerals and concentrate the polyphenol fraction by ultrafiltration. Finally, the liquid was spray dried. Phenolics concentration was estimated by comparison to a calibration curve prepared with chlorogenic acid using the Folin–Ciocalteu method [49]. The phlorotannins content of the extract was at least >20% chlorogenic acid equivalent (CAE). The two batches used in this study contained 24.1 and 29.7% CAE. The remaining constituents of the extract are mostly composed of fibers and minerals (iodine content = 63 and 64 mg/kg depending on the batch). The exact same extract was previously characterized [28]. The extract was dissolved to a final concentration of 0.1% (*m*/*v*) in sterile PBS and filtered through 0.2-μm filters thereafter. This concentration was chosen on the basis of previous work [24,50]. 

To compare our batches to the previously characterized extract, 1H-NMR spectra were acquired using an Oxford Instruments 600 MHz spectrometer equipped with a broad band inverse probe (Oxon, UK). The samples were extracted with 1 mL of solvent (deuterated methanol or deuterated water) and transferred to an NMR tube to be measured.

Cell culture: the A549 human lung cell line (ATCC CCL-185) was cultured in Dulbecco’s Modified Eagle Medium supplemented with 10% fetal bovine serum (FBS), 1% L-glutamine, 0.5% penicillin, and streptomycin. Confluent cultures in flasks were removed by trypsin incubation, and then cells were seeded at 80,000 cells/mL in 96-well microplates (100 µL/well) and kept at 37 °C for 24 h. After 24 h, cells were preincubated with the brown algae extract at 0.1% for 20 min and then incubated with B[a]P for 48 h or 72 h.

B[a]P stock solution at 40 mM was prepared in DMSO. This stock solution was diluted in culture medium containing 2.5% FBS to obtain concentrations of 1, 10 and 40 μM. Maximal final concentration of DMSO on cells was 0.1% (*v*/*v*).

Cytochrome P450 activity: EROD activity is a biomarker of exposure to polycyclic aromatic hydrocarbons and provides evidence of induction of cytochrome P450 (CYP) 1A1 and 1B1 [51,52]. After B[a]P incubation, a 2 μM solution of EROD was distributed in wells and the microplate was placed at 37 °C for 6 h. The fluorescence signal was then scanned (λexc = 535 nm, λem = 600 nm) using a microplate reader (Spark, Tecan, Männedorf, Switzerland).

P2X7 receptor activation: YO-PRO-1™, a fluorogenic probe, enters cells through P2X7 receptor activation-induced pores and emits fluorescence when it binds DNA [53,54]. Following our validated protocol, a 2 μM YO-PRO-1™ solution was distributed in wells and the microplate was placed at room temperature away from light for 10 min [55]. The fluorescence signal was then scanned (λexc = 485 nm, λem = 530 nm) using a microplate reader (Spark, Tecan). BBG was used as a specific P2X7 receptor antagonist, as previously described [56,57].

Filament actin arrangement: F-actin was stained with ActinGreen™ 488 ReadyProbes^®^ Reagent (Thermo Fisher Scientific, R37110). The cells were fixed in PFA 4% and permeabilized with Triton X-100 0.1%. A solution containing 2 drops of ActinGreen™ per mL of culture media was prepared, then the cells were incubated with the solution for 30 min. After washing, the cells were observed under fluorescence microscopy and pictures were taken (EVOS FL, Thermo Fisher Scientific).

E-Cadherin expression: after fixation in PFA 4% and permeabilization using Triton X-100 0.1%, the cells were incubated with rabbit anti-E-cadherin antibody (1/100, Thermo Fisher reference PA5-80457) diluted in PBS containing 1% of bovine serum albumin and 0.1% of Tween 20 overnight at 4 °C. After wash, the cells were incubated with Alexa 488-conjugated mouse anti-rabbit secondary antibody (Thermo Fisher) for 2 h at 4 °C. Cells were observed under fluorescence microscopy (EVOS FL) and pictures were taken.

Reactive oxygen species (ROS) production: once inside the cell, the fluorescent probe H2DCF-DA is cleaved by endogenous esterases and can no longer pass out of the cell. The de-esterified product becomes the fluorescent compound 2,7-dichlorofluorescein after oxidation by reactive oxygen species. After B[a]P incubation, the cells were incubated for 20 min with a 20 μM H2DCF-DA solution. Fluorescence detection (λexc = 485 nm, λem = 535 nm) was then undertaken with a microplate fluorometer (Spark, Tecan).

Statistical analysis: statistical analysis was performed using Prism software (version 6, GraphPad Software, La Jolla, CA, USA). A one-way analysis of variance for repeated measures followed by a Dunnett’s test were used to compare B[a]P incubation with control (*p*-value expressed as follows: *) and a t-test was used to compare results in the presence of the brown algae extract or BBG with results in the absence of the extract (*p*-value expressed as follows: $). 

## Data Availability

The data presented in this study are available on request from the corresponding author.

## Supplementary Materials

The following are available online at https://www.mdpi.com/1660-3397/19/1/34/s1, Figure S1: 1H-NMR spectrum of the filtered algal extract in deuterated methanol. Green spectra: batch #1, black spectra: batch #2; Figure S2: 1H-NMR spectrum of the filtered algal extract in deuterated water. Green spectra: batch #1, black spectra: batch #2.
