# Supplementary material for: In Vitro Chemopreventive Potential of Phlorotannins-Rich Extract from Brown Algae by Inhibition of Benzo[a]pyrene-Induced P2X7 Activation and Toxic Effects
Source: Mar Drugs. 2021 Jan 14;19(1):34. doi: 10.3390/md19010034 (PMC7828825; doi:10.3390/md19010034)
Supplement: Supplementary file 1 [file marinedrugs-19-00034-s001.pdf]

Supplementary Materials: ***In vitro* chemopreventive potential of phlorotannins-rich extract from brown algae by inhibition of benzo[a]pyrene-induced P2X7 activation and toxic effects**

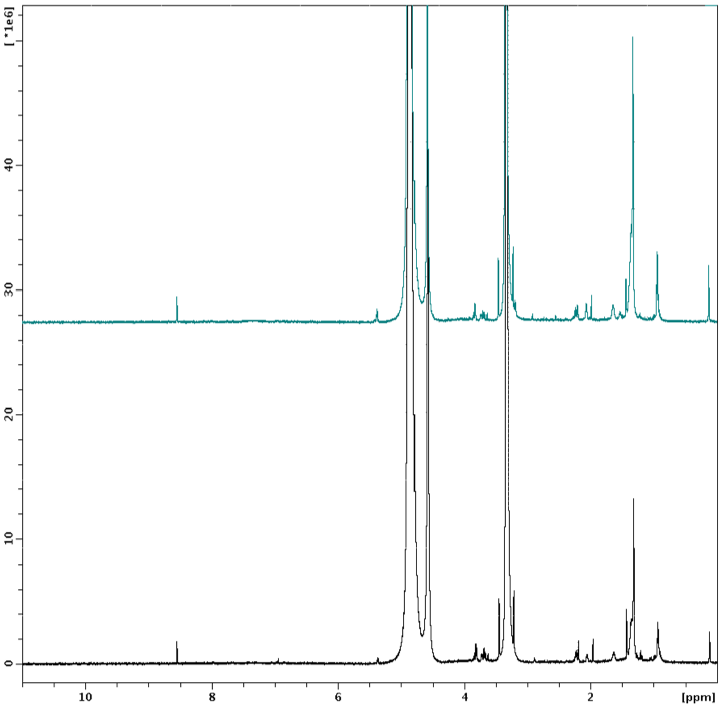

Figure S1: ^{1}H-NMR spectrum of the filtered algal extract in deuterated methanol. Green spectra: batch #1, black spectra: batch #2.

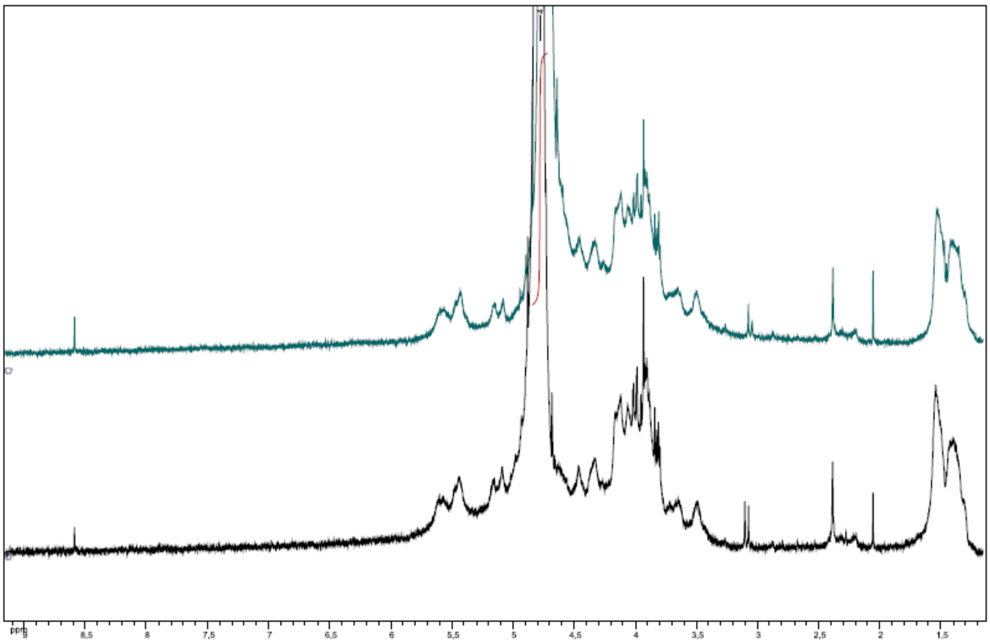

Figure S2: ^{1}H-NMR spectrum of the filtered algal extract in deuterated water. Green spectra: batch #1, black spectra: batch #2.
